# Inter- and intra-day test-retest reliability of the Cosmed Fitmate Pro^TM^ indirect calorimeter for resting metabolic rate

**DOI:** 10.1186/1550-2783-11-S1-P46

**Published:** 2014-12-01

**Authors:** Bill Campbell, Gina Zito, Ryan Colquhoun, Nic Martinez, Courtney St Louis, Mallory Johnson, Laura Buchanan, Matt Lehn, Yasmin Smith, Brad Cloer, Kathryn Raines

**Affiliations:** 1 University of South Florida, Tampa, Florida, USA

## Background

In order to make objective and intelligent decisions regarding caloric intake for fat loss/physique enhancement, one’s resting metabolic rate should be measured. It is important that the device used to measure resting metabolic rate be valid and reliable. Therefore, the purpose of this study was to establish the inter- and intra-day test-retest reliability of the Cosmed Fitmate ProTM indirect calorimeter for resting metabolic rate (RMR).

## Methods

34 participants (18 males [27 ± 9.5 years, 176.3 ± 7.0 cm, 85.5 ± 14.8 kg, 27.4 ± 4.1 BMI] and 16 females [31 ± 13 years, 163.4 ± 5.8 cm, 61.1 ± 12.3 kg, 22.9 ± 4.6 BMI]) volunteered to have their RMR measured a total of three times on two different days. On day 1, two 15-minute RMR tests were completed in back to back fashion. On day 2 (occurring within 7 days of day 1), another 15-minute RMR test was conducted. All RMR tests were conducted after an overnight fast in a rested condition. Reliability of RMR was evaluated using Pearson’s correlation, intraclass correlation coefficients (ICC), standard error of the measurement (SEM), and standard error of the measurement as a percentage of the mean (SEM%). Systematic error was examined using a one-way repeated measures ANOVA. Data was analyzed using SPSS version 22.0. Consent to publish the results was obtained from all participants.

## Results

Average RMR values for each test are shown in Table 1 below. The intra-day RMR Pearson correlation was r = 0.96 (p < 0.01) and the inter-day RMR Pearson correlation was r = 0.90 (p < 0.01). Intra-day RMR ICC was 0.981 and the inter-day RMR ICC was 0.946. Intra-day RMR SEM (SEM%) was 70.62 (4.13%) kilocalories. Inter-day RMR SEM (SEM%) was 116.9 (6.89%) kilocalories.

**Table 1:** Average Resting Metabolic Rate Values for Each Assessment

	Day 1 – RMR Test #1	Day 1 – RMR Test #2	Day 2 – RMR Test	ANOVA
Resting Metabolic Rate	1,711 ± 373 kcals	1,703 ± 370 kcals	1,679 ± 356 kcals	p = 0.460

## Conclusion

The Cosmed Fitmate ProTM indirect calorimeter is a reliable instrument for measuring resting metabolic rate in non-overweight males and females. The instrument demonstrated test-retest reliability both within the day and between two different days. Relative consistency was acceptable with ICC values of 0.981 and 0.946 for intra and inter-day measures, respectively. Absolute consistency was also acceptable with SEM values (expressed as a percentage of the mean) of 4.13% and 6.89% for intra and inter-day measures, respectively. Further, the ANOVA results indicated no systematic error among tests and days.

